# FAM188B enhances cell survival via interaction with USP7

**DOI:** 10.1038/s41419-018-0650-6

**Published:** 2018-05-24

**Authors:** Eun-Seok Choi, Hanna Lee, Jee Young Sung, Chang-Hun Lee, Hyonchol Jang, Kyung Tae Kim, Yong-Nyun Kim, Hyoung-Pyo Kim, Sung-Ho Goh

**Affiliations:** 1 Research Institute, National Cancer Center, Goyang, 10408 Republic of Korea; 2 Department of Environmental Medical Biology, Institute of Tropical Medicine, Yonsei University College of Medicine, Seoul, 03722 Republic of Korea

## Abstract

We have previously reported that *FAM188B* showed significant differential exon usage in cancers (NCBI GEO GSE30727), but the expression and function of *FAM188B* is not well characterized. In the present study, we explored the functions of *FAM188B* by a knockdown strategy, using siRNAs specific for *FAM188B* in colon cancer cell lines. *FAM188B* is a novel gene that encodes a protein that is evolutionarily conserved among mammals. Its mRNA has been found to be highly expressed in most solid tumors, including colorectal cancer. *FAM188B* knockdown induced cell growth inhibition due to an increase in apoptosis in colon cancer cell lines. Interestingly, siFAM188B treatment induced the upregulation and activation of p53, and consequently increased p53-regulated pro-apoptotic proteins, PUMA and BAX. Proteomic analysis of FAM188B immunocomplexes revealed p53 and USP7 as putative FAM188B-interacting proteins. Deletion of the putative USP7-binding motif in FAM188B reduced complex formation of FAM188B with USP7. It is noteworthy that FAM188B knockdown resulted in a decrease in overall ubiquitination in the p53 immunocomplexes, as well as p53 ubiquitination, because USP7 is involved in p53 deubiquitination. FAM188B knockdown inhibited both colony formation and anchorage-independent growth in vitro. In addition, FAM188B knockdown by siRNA reduced tumor growth in xenografted mice, with an increase in p53 proteins. Taken together, our data suggest that FAM188B is a putative oncogene that functions via interaction with USP7. Therefore, control of FAM188B could be a possible target to inhibit tumor growth.

## Introduction

Colorectal cancer (CRC) is the third most prevalent cancer worldwide and is a major contributor to cancer mortality^1^. CRC is heterogeneous disease, biologically classified into three major groups according to their molecular characteristics. The first is the chromosomal instable group, which accumulates mutations in specific oncogenes and tumor-suppressor genes. The second class is the microsatellite instability group, which leads to genetic hyper mutation, and the third is distinguished by CpG island methylation^2^. In addition, large-scale genomic studies have been conducted to advance our understanding of CRC at a molecular level, including The Cancer Genome Atlas analysis of 276 colon cancer patients^3^. Many critical pathways contribute to the development of CRC, including APC, WNT, RAS-MAPK, PI3K, TGF-β, TP53, and DNA mismatch repair^3^. However, despite these efforts, there is still lack of detailed characterization for low to intermediate frequency mutations or novel candidates.

Programmed cell death inhibits the development of cancer naturally through apoptosis of abnormal cells, but cancer develops when this mechanism is disrupted^4^. Typically, when chromosomal abnormality occurs, the expression of tumor-suppressor P53 is increased, leading to apoptosis of the cells^5^. Regulation of p53 is controlled by various post-translational modifications. The ubiquitin-proteasome system (UPS) is the main pathway for controlling protein integrity, and is central to the regulation of many cellular functions, notably including cell survival and death^6,7^. Ubiquitination is a remarkably complex, specific, three-enzyme (E1-E2-E3) cascade that utilizes 2 E1, 10 E2, and hundreds of E3 ubiquitin ligases^8^. Deubiquitinases (DUB, ubiquitin isopeptidase) are UPS components that catalyze removal of an ubiquitin moiety from poly-ubiquitin chains^6^; the human genome encodes 98 DUB genes classified into six families^9^. Thus, the dynamic and combinatorial interactions between ubiquitination and deubiquitination set the threshold for apoptotic signaling^10^. For example, the E3 ubiquitin ligase MDM2 ubiquitinates the tumor-suppressor p53, and DUBs, such as ubiquitin-specific proteases USP2a, USP7, USP10, USP22, and USP42, are involved in regulating the stability of p53 and MDM2 by removing ubiquitin moieties^6,11–13^. However, what determines whether p53 or MDM2 is the primary USP substrate is not known^10,14^.

A substantial proportion of genes (59%) in the human genome are reported as “hypothetical” and are annotated as being of “unknown function”^15^. Hypothetical proteins are predicted from nucleic acid sequences and their existence has not been experimentally proven. Another feature of the hypothetical protein is that it has low identity compared to known proteins^16^. However, despite their hypothetical status, which can be an obstacle to investigations of their expression patterns and potential functions in cellular pathways, such genes are often expressed to varying degrees in disease and are therefore biomedically relevant^17^. Thus, excluding “unknown” or “hypothetical” genes from analyses of candidate targets removes the opportunity to explore unprecedented molecular mechanisms that may be involved in clinically significant pathological dysfunctions. Recently, a hypothetical protein, FAM63A, was characterized as a new DUB family member, and the analysis of evolutionarily conservation among human genomes identified FAM63B as a homolog, and listed FAM188A and FAM188B as evolutionarily distant members^18^.

In our previous study, FAM188B showed significant differential exon usage in cancers (NCBI GEO GSE30727)^19^, but the expression and function of *FAM188B* had not yet been characterized. However, public database search revealed FAM188B was differentially expressed in many cancer types, and CRC showed significantly elevated expression in tumor. Here, we provide the first data that FAM188B is a genuine gene that overexpressed in CRC. In addition, we show that it functions in sustaining cell survival in vitro, and regulates growth in vivo. Our analyses revealed FAM188B intervenes in p53 stability control through interactions with USP7.

## Results

### Upregulation of FAM188B expression in colorectal cancers

**Fig. 1:**
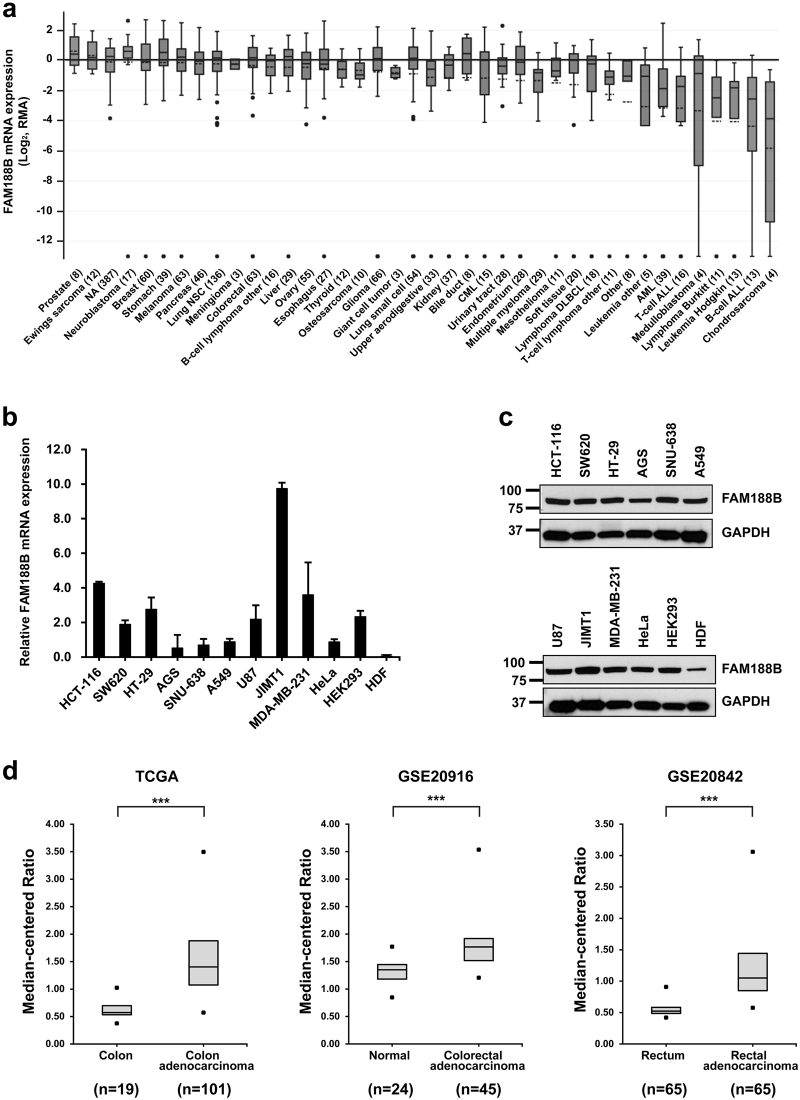
FAM188B expression levels in public database and validation in cell lines. **a** *FAM188B* mRNA expression levels in CCLE database. Box-and-whisker plots show the distribution of mRNA expression for each cancer type. Line in the box indicates median and dashed line indicates mean. **b** *FAM188B* mRNA levels were measured in cancer cell lines (HCT-116, SW620, HT-29, AGS, SNU-638, A549, U87, JIMT1, MDA-MB-231, and HeLa) and non-cancer cell lines (HEK-293 and HDF). Error bar indicates S.D. **c** Proteins (25 μg) from indicated cell lines were subjected to western blotting analysis using anti-FAM188B antibody. GAPDH was used as a loading control. Similar results were observed in three independent experiments. **d** *FAM188B* expression comparison between normal and tumor patient tissues using TCGA, GSE20916, and GSE20842 data set. Upper and lower dots indicate the maximum and minimum values. The middle line in the box indicates median (***p < 0.001)

Our previous study indicated that *FAM188B* has significant differential exon usage and differential expression in gastric cancer tissues^19^. To explore whether *FAM188B* expression is altered in other cancers, we first searched messenger RNA (mRNA) expression profiles of cancer cell lines using the Cancer Cell Line Encyclopedia (CCLE) database^20^. Most solid tumor cell lines, including CRC, showed a higher mRNA expression level of *FAM188B* than tumors from lymphoma, leukemia, and B-cell or chondrosarcoma (Fig. 1a). To verify *FAM188B* mRNA expression, we carried out quantitative real-time PCR (qRT-PCR) using various cancer and non-cancerous cell lines (Fig. 1b). *FAM188B* mRNA expression was detected to different levels in all cell lines tested, including CRC cell lines. To determine its endogenous protein expression, we generated a polyclonal antibody against FAM188B. Western blot analysis revealed that FAM188B protein was expressed in all cell lines (Fig. 1c). FAM188B protein levels appeared to be relatively low in human dermal fibroblasts (HDFs), which was correlated with low mRNA levels. As FAM188B expression at both mRNA and protein level were substantial in the CRC cell lines (HCT-116, SW620, and HT-29), we further explored public datasets regarding FAM188B expression in CRC patients. *FAM188B* mRNA expression was highly increased in multiple CRC datasets (TCGA colorectal 2, *p* = 2.48 × 10^−16^; GSE20916, *p* = 6.59 × 10^−7^; GSE20842, *p* = 2.68 × 10^−26^) from Oncomine database^21^ (Fig. 1d).

### FAM188B downregulation leads to cell death in vitro

**Fig. 2:**
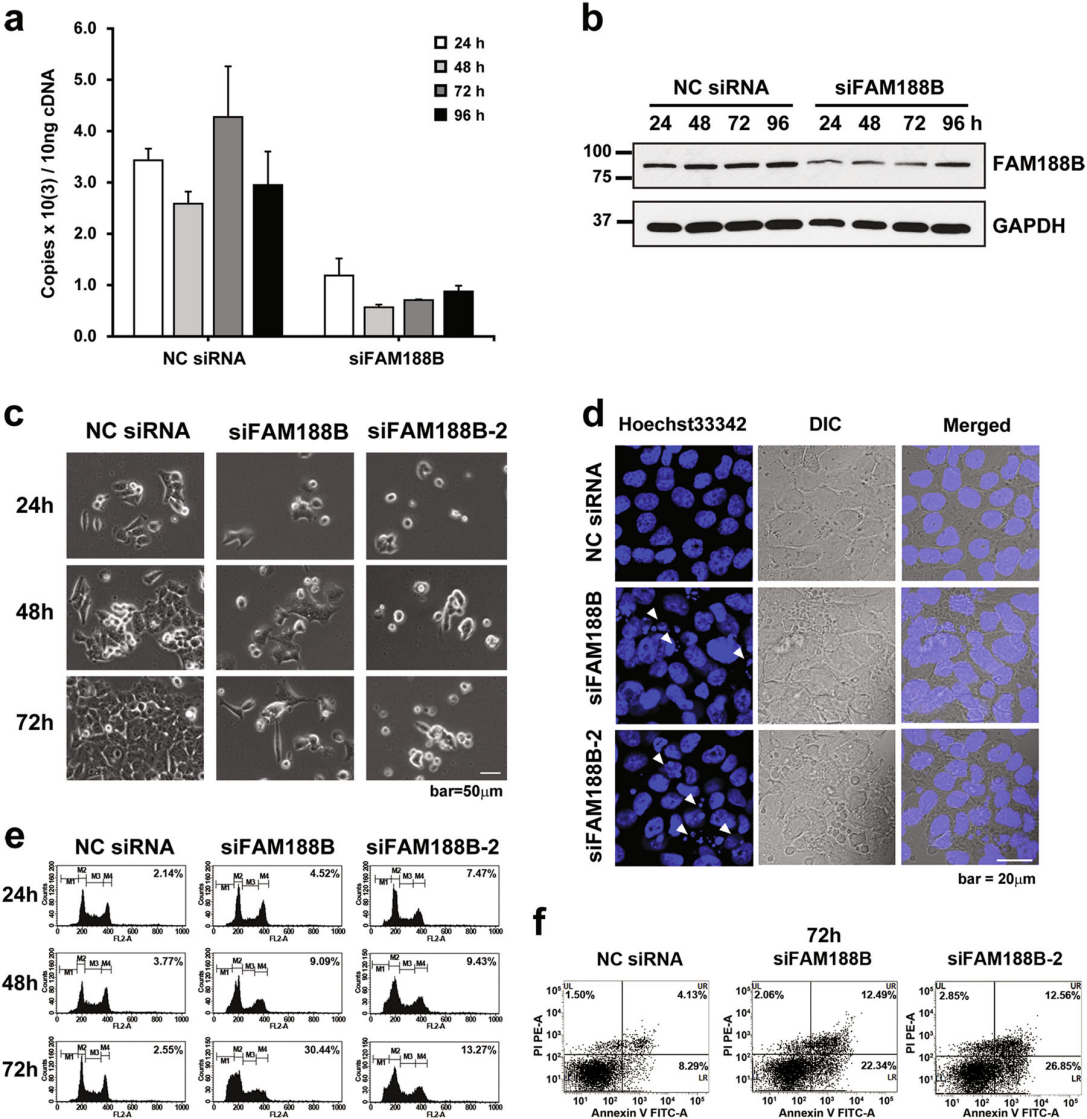
Effects of FAM188B knockdown on cell death. **a**, **b** HCT-116 cells were transfected with either NC siRNA or siFAM188B for indicated times. The mRNA levels and protein levels of FAM188B were measured by qRT-PCR (**a**) and western blotting (**b**), respectively. **c**–**f** HCT-116 cells were transfected with either NC siRNA, siFAM188B, or siFAM188B-2 and images for cell growth (**c**) and nuclei fragmentation (**d**) were taken at the indicated times. White arrow heads indicate nuclei fragmentation (scale bar = 20 μm) (**d**). Cells were also processed for cell-cycle analysis by flow cytometry; dead cell population: sub-G0/G1 is indicated in (%) (**e**). For apoptosis analysis, after 72 h of siRNA transfection cells were stained with annexin V/PI, followed by flow cytometry (**f**) (PI-stained cells apoptosis population: UR and UL)

To understand the function of *FAM188B*, we knocked down FAM188B expression using *FAM188B*-specific siRNA in the colon cancer cell lines HCT-116, HT-29, and SW620. Transfection of cells with siFAM188B decreased *FAM188B* mRNA expression from 24–96 h as determined by qRT-PCR (Fig. 2a and Supplementary Figures 3a and 4a), whereas negative control (NC) siRNA had little effect on FAM188B expression. Correspondingly, *FAM188B* mRNA levels decreased. FAM188B protein levels were also reduced by siFAM188B, as determined by western blot (Fig. 2b, Supplementary Figures 3b and 4b). To test the effects of *FAM188B* knockdown on cell growth, we employed two different siFAM188B RNAs (siFAM188B and siFAM188B-2). When FAM188B was knocked down by either siFAM188B or siFAM188B-2, HCT-116 cell growth decreased with time (Fig. 2c, Supplementary Figures 3c and 4c). A microscopic analysis of DAPI-stained cells also revealed that siFAM188B treatment increased brightly fluorescent and fragmented nuclei, which is indicative of apoptosis (Fig. 2d). As cell growth inhibition could occur either by cell-cycle arrest or cell death, we examined the effect of *FAM188B* knockdown on cell cycle. Interestingly, the sub-G0/G1 population increased in the siFAM188B-treated cells, which corresponds to apoptotic cells (Fig. 2e). In addition, *FAM188B* knockdown increased the population of annexin V/PI-stained cells (Fig. 2f). Apoptosis induced by siFAM188B was not limited to HCT-116 cells because compared with NC siRNA-treated cells, siFAM188B treatment also increased annexin V/PI-positive cells in other colon cancer cell lines, HT-29 and SW620 (Fig. 2f, Supplementary Figures 3d and 4d). All these data suggest that downregulation of FAM188B expression in colon cancer cells leads to apoptosis.

### FAM188B silencing activates p53 and its downstream pathway

**Fig. 3:**
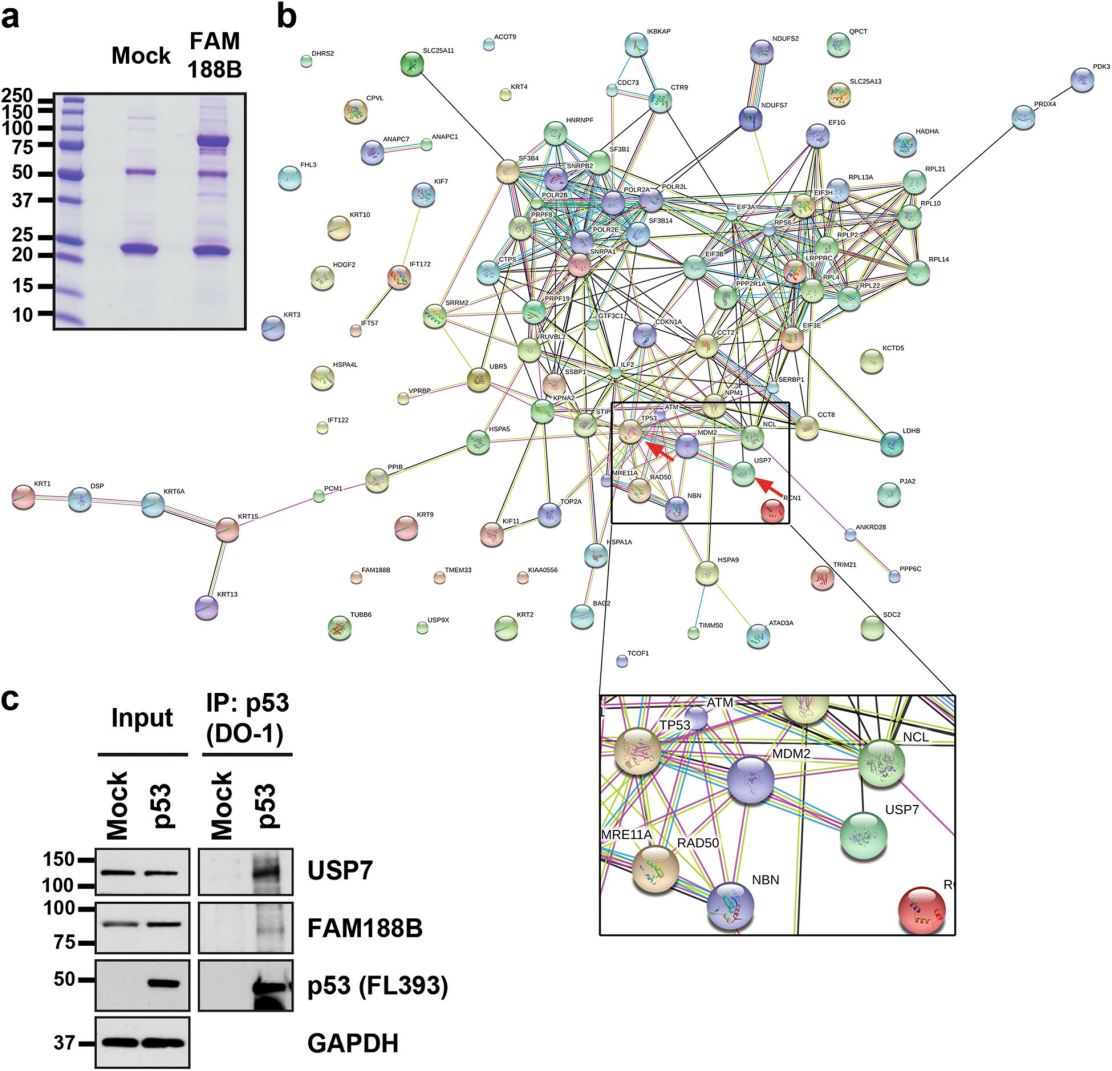
Analysis of FAM188B-interacting proteins. **a** FLAG-tagged FAM188B was overexpressed in HEK-293 cells and immunoprecipitated complexes of FLAG-tagged FAM188B were resolved on SDS-PAGE gels. Image of Coomassie-blue staining of the gel is shown. **b** The resolved proteins in (**a**) were processed for LC/MS-MS analysis and searched against String-DB to see the interactions in “Evidence View.” Black arrow indicates interacting protein separately confirmed by immunoprecipitation. **c** Validation of FAM188B interaction to USP7 and p53 by immunoprecipitation of HEK-293 cell lysate with p53 (DO-1) antibody and immunoblotted with indicated antibodies

To investigate the mechanism of FAM188B involvement in cell death, we searched for potential cellular FAM188B-binding partners by liquid chromatography/mass spectrometry (LC/MS)-MS (Fig. 3a). Mass peaks from MS searches against the database identified a total of 104 unique proteins bound to FAM188B. These proteins were analyzed using the DAVID server^22^ and String-DB^23^ to cluster proteins according to their involvement in intracellular biological processes and evidence-based interacting groups, respectively. As expected, based on results obtained by FAM188B downregulation, many FAM188B-binding proteins were involved in cell-cycle regulation and apoptosis (Supplementary table 2). Notably, clusters on the interaction map of FAM188B-binding proteins revealed several clusters including the tumor-suppressor proteins p53 as well as USP7 (Fig. 3b, arrows). Interestingly, when we immunoprecipitated p53 proteins, we could observe that FAM188B as well as USP7 were in the p53 immunocomplexes (Fig. 3c), indicating that FAM188B forms a complex with p53.

**Fig. 4:**
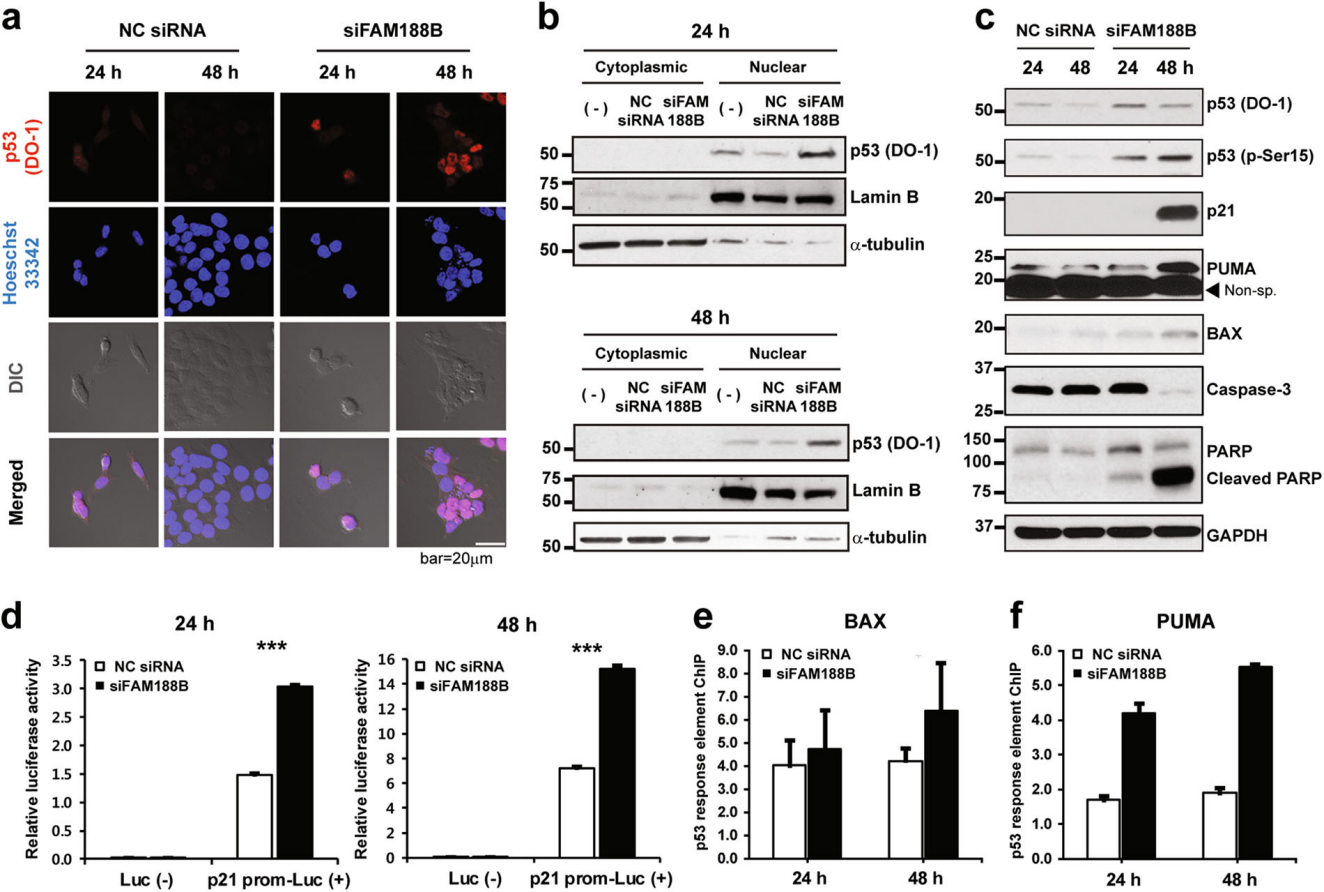
Effects of FAM188B knockdown on p53 activation. **a** HCT-116 cells were transfected with either NC siRNA or siFAM188B for indicated times. Cells were then stained using anti-p53 antibody and Hoechst33342 for nucleus followed by confocal microscopy analysis (scale bar = 20 μm). **b** After treatment of cells as in (**a**), cells were fractionated and cytosol and nuclear fractions were subjected to western blotting using indicated antibodies. PARP and α-tubulin were used as nuclear and cytoplasmic markers, respectively. **c** After treatment of cells as in (**a**), cellular proteins were analyzed by western blotting using indicated antibodies. **d** Transcriptional activation of the p21 promoter, a target of p53, was measured using a dual luciferase assay. ***p < 0.001. The p53-responsive element of *BAX* (**e**) and *PUMA* promoter regions (**f**) was measured by ChIP qRT-PCR assays. Three independent ChIP qRT-PCR assays of *BAX*-p53RE levels and *PUMA*-p53RE were normalized to input control, respectively

Because p53 is an important regulator for cell death control, we further examined a possible association of p53 in the siFAM188B-induced apoptosis in more detail. When cells were stained for p53, more p53 were localized in the nucleus in the siFAM188-treated cells than in the NC siRNA-treated cells (Fig. 4a). Consistent with these immunocytochemistry data, more p53 protein was detected in the nucleus fraction than in the cytosolic fraction when FAM188B was knocked down (Fig. 4b). Next, we tested whether p53 is activated in the *FAM188B* knocked-down cells by immunoblotting using antibodies that recognize Ser15-phosphorylated p53 (active form of p53)^24^. *FAM188B* knockdown increased p53 as well as Ser15-phosphorylated p53, and thus enhanced protein levels of p53-regulated genes, including p21, PUMA, and BAX, which are in the apoptosis pathway (Fig. 4c). To further test p53 activation transcriptionally, we performed promoter assay using p21 promoter-luciferase construct because p21 is known to be a p53 target gene^25^. siFAM188B treatment increased the transcriptional activity of p53 toward its downstream target p21 by approximately twofold (Fig. 4d). Moreover, chromatin immunoprecipitation (ChIP) assays using p53 antibodies showed increased binding of p53 to *BAX* (Fig. 4e) and *PUMA* promoters (Fig. 4f) in the siFAM188B-treated cells. These results suggest that FAM188B downregulation activates p53, and thus upregulates apoptosis-related genes, including BAX, and PUMA, and leads to cell death.

### FAM188B regulates p53 stability via interaction with USP7

**Fig. 5:**
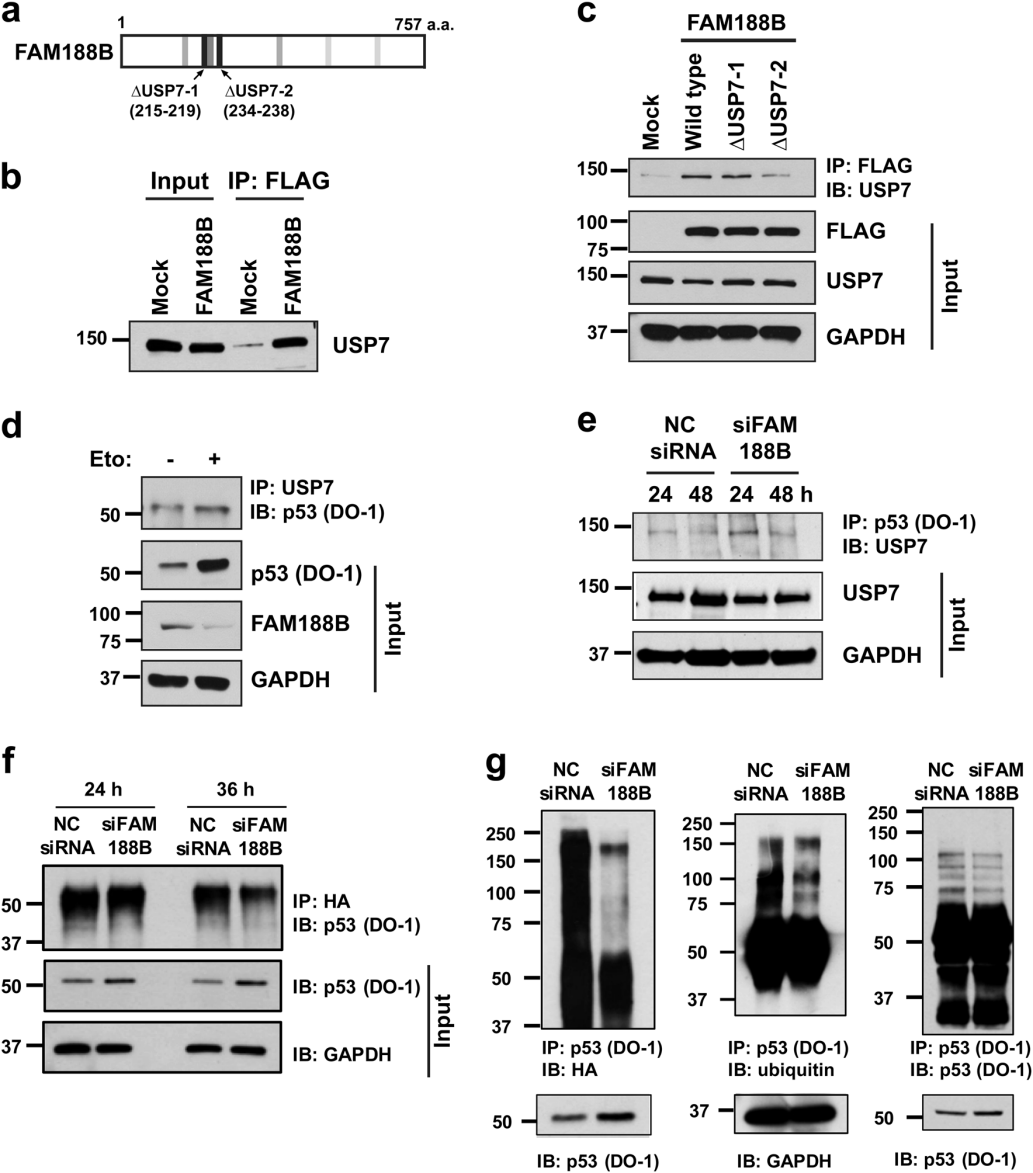
USP7 interacts with FAM188B and knockdown of FAM188B regulates p53 deubiquitination. **a** Predicted position of seven USP7-binding motifs were indicated on FAM188B protein. ΔUSP7-1 and -2 are the positions of deletion mutants to analyze FAM188B-USP7 interaction. **b** Co-immunoprecipitation of overexpressed FAM188B and USP7 in HCT-116 cell. **c** Verification of the interaction between FAM188B and USP7 using overexpression of wild-type and the predicted USP7-binding motif deleted FAM188B constructs. **d** Lysates from HCT-116 cells treated with or without etoposide (5 μM) were immunoprecipitated and immunoblotted with antibodies shown in the figure. **e** FAM188B silencing increased the interaction between USP7 and p53. Cell lysates from HCT-116 cells treated with NC siRNA or siFAM188B for 24 and 48 h were immunoprecipitated with anti-p53 antibody and immunoblotted with anti-USP7 antibody. **f**, **g** Knockdown of FAM188B reduces ubiquitinated p53. **f** HA-tagged ubiquitin (5 μg) was transfected to HCT-116. Cell lysates from HCT-116 treated with NC siRNA or siFAM188B were immunoprecipitated by anti-HA antibody and immunoblotted with indicated antibodies. **g** Cell lysates from HCT-116 co-transfected with HA-tagged ubiquitin and NC siRNA or siFAM188B were immunoprecipitated by anti-p53 (DO-1) antibody and immunoblotted with indicated antibodies

To determine how *FAM188B* knockdown enhances p53 protein levels, we identified FAM188B-interacting proteins revealed by LC/MS-MS (Fig. 3b). As FAM188B silencing affects p53 levels, we tested whether USP7, a deubiquitinase, and p53-interacting proteins detected in LC/MS-MS experiments, can be detected as a FAM188B-binding protein. In accordance with this, the online protein domain prediction site, “Eukaryotic Linear Motif resources”^26^, also predicted the presence of a USP7-binding motifs in FAM188B (Fig. 5a and Supplementary Figure 1a). USP7 protein was detected in FAM188B immunoprecipitates in the cells with FAM188B overexpression (Fig. 5b), indicating a complex formation of FAM188B with USP7. To investigate which motif of FAM188B is responsible for its complex formation with USP7, either FLAG-tagged wild-type or mutant FAM188B with deletion of USP7-binding motif-1 (ΔUSP7-1) or USP7-binding motif-2 (ΔUSP7-2) were overexpressed in HCT-116 cells, followed by immunoprecipitation of exogenous FAM188B using anti-FLAG antibodies. USP7 was consistently found in the wild-type FAM188B immunocomplexes but USP7 levels decreased in the mutant FAM188B immunocomplexes (ΔUSP7-2), suggesting that USP7-2 motifs of FAM188B are important for its complex formation with USP7 (Fig. 5c). Next, to examine whether USP7 interacts with p53, we performed a co-IP assay after etoposide treatment to increase p53 expression. Etoposide treatment resulted in an increase in p53 and a decrease in FAM188B. P53 was found in the USP7 immunocomplexes and this complex formation increased with etoposide treatment (Fig. 5d). Next, we tested whether FAM188B levels affect complex formation of USP7 and p53. A basal level of USP7 was detected in the p53 immunocomplexes. However, interestingly, this complex formation appeared increased in the siFAM188B-treated cells after 24 h, and it became weaker at 48 h although the endogenous USP7 protein expression was not changed by FAM188B silencing (Fig. 5e).

To test whether FAM188B is involved in p53 deubiquitination, we examined the change in the level of ubiquitinated p53 after *FAM188B* knockdown in the cells expressing HA-tagged ubiquitin. Compared with NC siRNA-treated cells, less p53 was detected in the HA-immunoprecipitated complexes from siFAM188B-treated cells (Fig. 5f). In addition, the level of HA-tagged ubiquitin decreased in the p53 immunocomplexes from siFAM188B-treated cells compared with those of NC siRNA-treated cells. A lower level of endogenous ubiquitinated p53 was also detected in the p53 immunocomplexes from siFAM188B-treated cells than those of NC siRNA-treated cells (Fig. 5g).

### FAM188B knockdown inhibits tumor growth in vivo

**Fig. 6:**
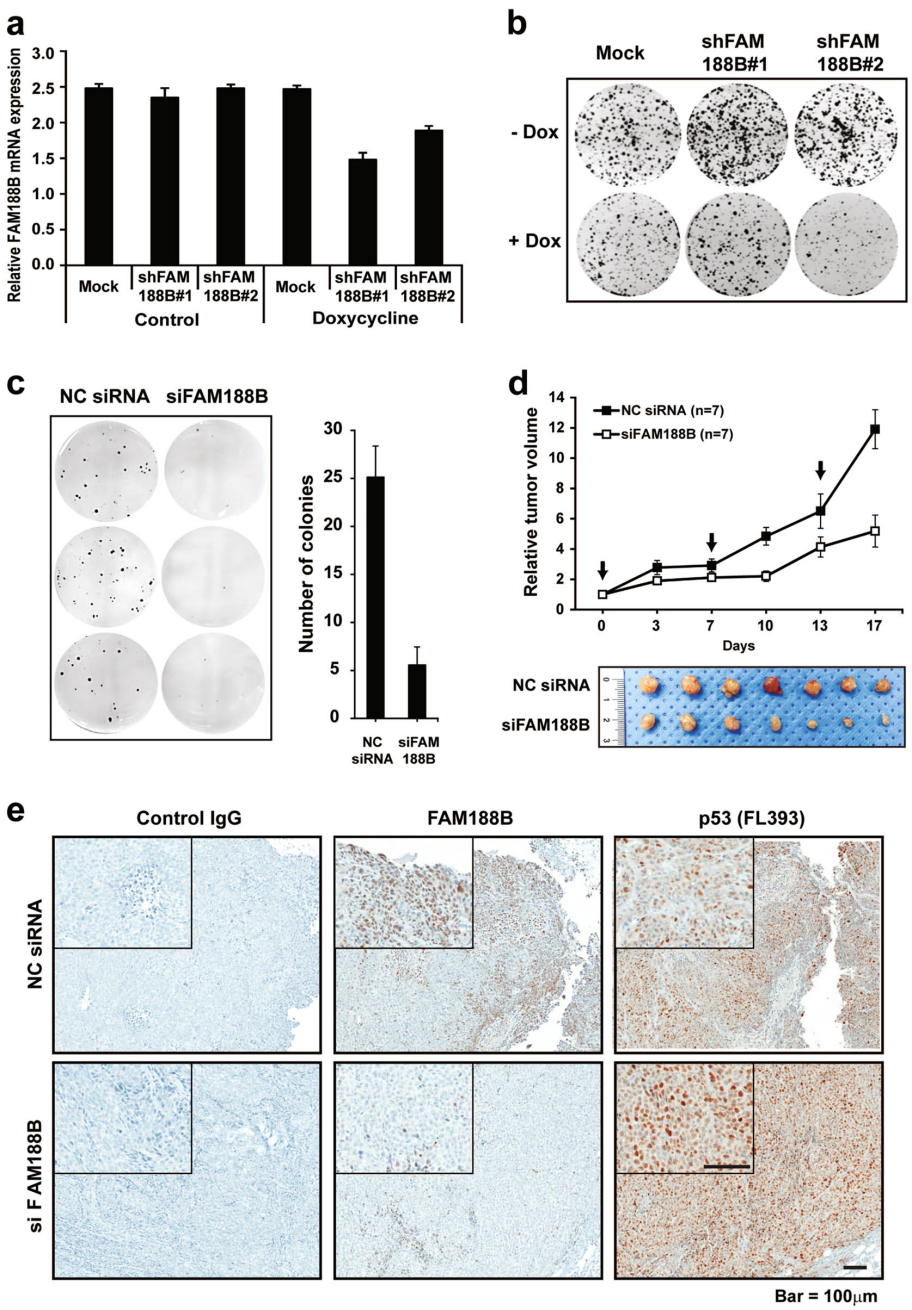
Oncogenicity of FAM188B confirmed in vitro and in vivo. **a** mRNA expression in HCT-116 shRNA stable cell lines was measured by quantitated real-time PCR. Doxycycline induces shRNA in HCT-116. Error bar indicates S.D. **b** shRNA stable HCT-116 cells were treated with doxycycline to induce shRNA expression and incubated 7 days to form colonies and stained with crystal violet. The number of colonies of Dox (+) is significantly reduced compared to Dox (−). **c** HCT-116 cells (5.0 × 10^3^) treated with NC siRNA or siFAM188B was cultured in soft agar for 18 days. The colonies were stained with INT solution. All experiments were performed triplicate. **d** HCT-116 cells (2.0 × 10^6^) were injected into the nude mice (*n* = 7) subcutaneously and tumor growth was monitored twice a week. When tumor size reached 30 mm^3^, siRNAs were treated a week for 3 weeks. Black arrow indicates the siRNA treatment. Tumor size was measured at indicated time points (representative images of tumors; NC siRNA (top lane) and siFAM188B (bottom lane)). **e** Immunohistochemistry of FAM188B and p53 protein detected from tumor tissues from mice using control IgG, anti-FAM188B, and anti-p53 (FL393) antibodies (scale bar = 100 μm)

To further investigate the effect of FAM188B downregulation on tumor growth, we established a shFAM188B-inducibile HCT-116 stable cell line. Doxycycline induced *FAM188B* short hairpin RNA (shRNA), which led to FAM188B downregulation (Fig. 6a). FAM188B shRNA expression reduced the number of colonies (Fig. 6b). Next, we examined the effects of *FAM188B* knockdown on anchorage-independent colony formation by soft agar assay. When HCT-116 cells were treated with siFAM188B, anchorage-independent colony formation was significantly decreased (Fig. 6c). These data indicated that FAM188B might have an oncogenic function. To test the effect of FAM188B silencing on tumor growth in vivo, HCT-116 cells were xenografted into BALB/c nude mice, and *FAM188B* small interfering RNA (siRNA) was delivered by electroporation. Tumor volume was regularly measured after treatment with *FAM188B* siRNA. The growth of siFAM188B-treated tumors was significantly reduced from the first week compared to that of NC siRNA-treated tumors (Fig. 6d). When the tumors were removed from the killed mice, siFAM188B-treated tumors were smaller than the NC siRNA-treated tumors (Fig. 6d). To verify the *FAM188B* knockdown in the xenografted tumors, tumor tissues were processed for immunostaining using anti-FAM188B antibodies. Immunohistochemistry analysis showed that FAM188B protein levels reduced, while p53 protein levels increased, in the siFAM188B-treated tumors (Fig. 6e). These data indicate that FAM188B expression is important for tumor growth in vitro and in vivo. Taken altogether, our data indicate that FAM188B has a critical oncogenic effect, possibly via enhancing p53 ubiquitination and thus p53 downregulation. Therefore, targeting FAM188B could be a good strategy to control tumor growth.

## Discussion

Cancer progression is the result of various biological processes, and may also involve unexplored functions of novel/hypothetical genes, and/or variants of known genes. Despite the tremendous effort devoted to analyze available genomic information, as much as 59% of human genes were annotated as “hypothetical” when the human genome was first reported^15,27^, and 24–31% of entrez/ensemble database entries are currently annotated as “uncharacterized”^17^. Our previous study, profiling exon usage in gastric cancer, identified *FAM188B* as a gene that was significantly alternatively spliced between gastric cancer and adjacent normal tissue. Until now, *FAM188B* was annotated as a hypothetical gene for which the only evidence for function was its transcript; no confirmation of its expression at the protein level had yet been provided.

Before we characterized the function of FAM188B, we first analyzed the evolutionary conservation of the protein sequences among species, because a biologically important gene would be preserved in the genome, even if its function is not known. We obtained 15 orthologous protein sequences to FAM188B from bony fish to chimpanzee from GenBank and aligned these sequences with human FAM188B (Supplementary Figure 1a) and compared the evolutionary distance among them (Supplementary Figure 1b). All of the homologs showed high similarity, and the sequence similarity of DUF4205 (domain of unknown function: IPR025257)^28^ among mammalian FAM188B homologs was >76% (Supplementary Figure 1a). According to the cladogram, human FAM188B was closely related with the other orthologues from primates such as *Pan troglodytes* (chimpanzee), *Pongo abelii* (orangutan), but far from amphibian *Xenopus tropicalis* (Supplementary Figure 1b). These results suggest that FAM188B might be conserved through the evolutionary lineage of the vertebrates for a biologically indispensable function.

We first determined whether this gene is a protein-coding gene or a non-coding gene. The mature mRNA sequence of *FAM188B* from the colon cancer cell line HCT-116 showed a poly-A signal sequence in its 3′ UTR (data not shown), suggesting that this gene could indeed encode a protein. This evidence led us to generate a rabbit polyclonal antibody to detect the FAM188B protein. Using the results of anti-FAM188B-specific antibody, we could detect endogenously or overexpressed FAM188B protein by immunoblotting and immunohistochemistry (Supplementary Figure 2). Furthermore, as revealed by transcriptome profiles of the public databases including the CCLE, Oncomine, and NCBI GEO^29^, the expression of *FAM188B* was significantly enhanced in tumors at the mRNA level. These results suggest that *FAM188B* has important housekeeping functions in maintaining cell viability.

Having obtained evidence of endogenous expression, we explored the functional implications of elevated *FAM188B* expression in tumors by suppressing translation of its mRNA using a siRNA approach. This loss-of-function study showed that knockdown of *FAM188B* resulted in a significant increase in apoptotic cellular phenotypes, including accumulation of a sub-G0/G1 cell-cycle population, fragmented nuclei, and an increase in the dead cell population. These phenotypic changes were also observed in other cancer type cell (AGS, a gastric cancer cell; Supplementary Figure 5), thus providing strong evidence of the potential role of *FAM188B* as an oncogene or regulator of oncogenic pathways. However, identifying genuine interacting partners—a key to the known world of biological pathways—is crucial to understanding the working mechanisms of this unknown protein. The catalog of FAM188B-interacting proteins was defined through immunoprecipitation of FAM188B followed by LC/MS-MS analysis. A clustering of these proteins according to their gene ontology (GO) biological process showed that the major clusters were “protein translation” and “cell death/apoptosis,” followed by “chromatin regulation” and “RNA-binding proteins” (Supplementary Table 2). Among these biological processes, cell death and protein degradation categories were of interest, because knockdown experiments suggested that this protein might be involved in regulating cell survival. Thus, we verified the FAM188B-interacting proteins identified by mass spectrometry using co-immunoprecipitation, and then proceeded to characterize FAM188B functions related to cell death. Immunoprecipitation using an anti-p53 (DO-1) antibody confirmed the interaction of FAM188B with p53 (Fig. 3c). Intriguingly, GO analyses categorized several proteins into an “ubiquitin-dependent protein” group; among them was USP7 (also known as HAUSP), which clustered with p53 as a FAM188B-interacting protein (Fig. 3b). USP7 is known to stabilize p53 by deubiquitinating it, as well as its inhibitor MDM2^30,31^. As such, USP7 has come to be considered a therapeutic target in many cancers^6,13^. In breast cancer, TSPYL5 was reported to reduce p53 levels through physical interactions with USP7^32^. In addition to USP7, USP2a and USP10 are also involved in regulating p53 ubiquitination in different contexts^6^; thus, evidence supporting USP7 as the major p53 modulator is inconclusive. In our study, however, the amount of FAM188B-bound USP7 dramatically increased upon FAM188B overexpression in HCT-116 cells. Conversely, FAM188B downregulation increased unbound USP7 and this free USP7 may decrease the level of ubiquitinated p53. The fact that USP7 plays a pivotal role in deubiquitinating both p53 and MDM2, as noted above, seemingly undermines the interpretation that USP7 is a p53 stabilizer. This may be the reason why the decrease of ubiquitinated p53 in siFAM188B-treated cells was not extreme (Fig. 5g). However, the shorter half-life of MDM2 compared to p53, as a result of MDM2 self-ubiquitination^14,33^, may ultimately lead to cell death through accumulated p53. If a mediator exists that could be the answer to the questions “What determines whether p53 or MDM2 is the primary USP7 substrate?”^10^ and “What determines how USP7 is regulated?”^14^, FAM188B might be that mediator. In tumor cells, high levels of FAM188B would be predicted to function by eliminating p53 and keeping it at a low level to allow tumor cells to grow. Silencing of USP7 decreased the p53 level and increased ubiquitinated-p53. However, double silencing of USP7 and FAM188B restored the p53 level (Supplementary Figure 6a). Therefore, elevated expression of FAM188B in tumor tissues suggests that FAM188B would inhibit p53 activation and thereby provide tolerance against stressful conditions (Supplementary Figure 6b). On the other hand, many tumors have mutations in or deletion of p53 gene, therefore, the effect of elevated FAM188B may not be exclusive on the tumorigenesis in such tumors.

The most intriguing result in this study was confirming the tumorigenicity of FAM188B. The colony forming ability of FAM188B was significantly abolished by silencing its expression in vitro. Interestingly, in soft agar colony formation assays, a golden standard to determine cellular transformation and tumorigenicity^34^, FAM188B silencing showed dramatic reduction of anchorage-independent colony growth (Fig. 6b). Moreover, in vivo tumor growth assays also revealed that FAM188B led to a dramatic difference in tumor masses of xenografted HCT-116 in BALB/c nude mice. Accordingly immunohistochemical analyses revealed that FAM188B is a vital gene for proliferation or survival. Although additional studies with human tissue are also required to assess feasibility, these results suggest that FAM188B overexpression could be used as a predictive biomarker for cancer diagnosis, and suggests that inhibiting FAM188B activity could be utilized as the putative target of cancer progression. Although further studies are required to reveal how FAM188B expression is regulated, our findings clearly support the conclusion that FAM188B is an important regulator of p53 stability in growing cells.

In this study, the hypothetical protein FAM188B was revealed to be a genuine protein whose expression is significantly elevated in colon cancer cell lines. On the basis of FAM188B loss-of-function analyses, we suggest that FAM188B functions to sustain cell viability by decreasing p53 activation, through inhibition of the deubiquitinase USP7. This provides significant insight into the importance of FAM188B in sustaining cell survival, as well its use as a potential target in cancer therapy. Further research on the details of FAM188B regulatory mechanisms, as well conditions with p53 mutations, will increase our understanding of FAM188B as a potential therapeutic target.

## Materials and methods

### Datasets from the public databases

Datasets were obtained from Cancer Cell Line Encyclopedia (CCLE) database (https://portals.broadinstitute.org/ccle)^20^ for cancer cell lines. Datasets of colorectal adenocarcinoma with >2× of expression difference with *p*-value <0.001 between normal and tumor tissues were obtained from Oncomine (https://www.oncomine.org/)^21^.

### Cell lines

The human cell lines used in these studies (HCT-116, SW620, HT-29, AGS, SNU-638, A549, U87, JIMT1, MDA-MB-231, HeLa, HEK293, and HDF) were obtained from American Type Culture Collection (Manassas, VA, USA) or Korean Cell Line Bank (Seoul, Korea). All the cells were cultured with designated media (Corning, Manassas, VA, USA) supplemented with 10% fetal bovine serum (Corning) and 1× penicillin streptomycin (Invitrogen, Carlsbad, CA, USA) at 37 °C containing 5% CO_2_.

### Reverse-transcription polymerase chain reaction and quantitative RT-PCR

First-strand complementary DNA synthesis and qRT-PCR were performed as our previous report using a LC480 real-time PCR machine (Roche, Switzerland)^35^. Primers spanning two consecutive exons (Supplementary Table 1) were designed using Primer3 software (http://frodo.wi.mit.edu/primer3/)^36^.

### Antibodies for immunoblot and immunocytochemistry

For the detection of FAM188B, we generate polyclonal antiserum using FAM188B protein C-terminus peptides (722–738 a.a: TISEDTDNDLVPPLELC) as antigen (AbFrontier, Seoul, Korea) (Supplementary Figure 2). The affinity-purified serum was applied to immunoblots at 1:1000 dilutions in 5% skim milk/1× Tris-buffered saline (TBS). The following antibodies were used to detect other proteins in this study: FLAG M2 and β-actin from Sigma-Aldrich (St. Louis, MO, USA); PUMA, USP7, p21, BAX and PARP from Cell Signaling Technologies (Danvers, MA, USA), and p53 (DO-1), p53 (FL393), phospho-p53 (ser15), and α-tubulin from Santa Cruz Biotechnology (Dallas, TX, USA), and GFP (JL-8) from Clontech (Mountain view, CA, USA).

### Analyses of FAM188B expression knockdown

FAM188B expression was silenced by transfecting with *FAM188B*-specific siFAM188B (Qiagen, Germany) targeting the sequence 5′-CTGACCATTGACACCACCAA-3′, Allstar NC siRNA (Qiagen) at 5 nM using Lipofectamine RNAiMAX (Thermo Fisher Scientific, San Jose, CA, USA).

Cell cycle was analyzed by flow cytometry by staining with propidium iodide (Sigma-Aldrich). Nuclear fragmentation in siRNA-treated cells stained with Hoechst33342 (Sigma-Aldrich) was observed by confocal microscopy at ×400 magnification. Annexin V assays for the detection of apoptotic populations was carried out using a BD FITC annexin V apoptosis detection kit I (BD Pharmingen, San Jose, CA, USA) according to the manufacturer’s protocol.

### Colony forming assay and soft agar assay

Doxycycline inducible HCT-116 shRNA (shFAM188B#1: CTTTGGAAATACGGCTAACAA, #2: CAGATACTTTCTGGATCACTT) stable cell lines (2 × 10^3^) seeded in six-well plates and 1 μg/ml of doxycycline (Sigma-Aldrich) was treated for 1 week. Colony formation was analyzed by fixing with 4% formaldehyde and staining with crystal violet solution (Sigma-Aldrich).

Onto the base agar (0.8%) dispensed into the wells, siRNA-treated HCT-116 cells were seeded with top agar (0.3%). Cells were cultured for 3 weeks with medium change every 4–5 days, and stained with INT solution.

### Proteomic analysis and profiling of interacting partners

FAM188B-interacting proteins from HEK-293 cells transfected with pFLAG-FAM188B plasmid were immunoprecipitated with anti-FLAG-M2 agarose affinity gel (Sigma-Aldrich). Analyses were performed using an LTQ XL linear ion trap mass spectrometer (MS) system (Thermo Fisher Scientific). All MS/MS samples were analyzed using Proteome Discover software (version v.1.4; Thermo Fisher Scientific), set up to search the Uniprot database and IPI human database.

### Dual luciferase assay

HCT-116 cells were transfected with NC siRNA or siFAM188B with pGL2-p21-*luc* (Addgene, Cambridge, MA, USA) and pGL4.70*hRluc* (Promega, Madison, WI, USA) plasmids. After 24 and 48 h, cells were collected, and dual luciferase assays were performed according to the manufacturer’s protocol (Promega) and measured using a Victor^3^ reader (Perkin Elmer).

### Chromatin immunoprecipitation assay

Collected nuclei from fixed cells (1 × PBS/1% formaldehyde) were sonicated for 7.5 min (0.5 min on, 1 min off cycles) using a Bioruptor (Diagenode, Denville, NJ, USA). Fragmented genomic DNA was immunoprecipitated with anti-p53 antibody (DO-1), and purified by phenol/chloroform/isoamyl alcohol extraction. Amplification of *BAX* and *PUMA* genes was carried out using SYBR green master mix with p53-RE primers^37^ using LC480 PCR machine (Roche).

### p53 ubiquitination assay

HCT-116 cells were co-transfected with HA-Ubi plasmid and NC siRNA or siFAM188B. MG132 treated for 4 h before collection. One milligram of cell lysates were immunoprecipitated with anti-p53 (DO-1) antibody and immunoblotted with anti-HA antibody and vice versa. For the input control, 20 μg of lysates were loaded and immunoblotted with p53 (DO-1), ubiquitin, HA, and GAPDH antibodies.

### In vivo analysis of FAM188B silencing

HCT-116 (2.0 × 10^6^) cells were subcutaneously injected in 6-week-old male BALB/c nude mice (CAnN.Cg-Foxn1nu/CrljOri, Orient Bio, Korea). When the tumor size reached 30 mm^3^, Allstars NC siRNA or siFAM188B were injected into tumor via electroporation using NEPA21 Super Electroporator TypeII (Nepa Gene Co., Chiba, Japan) every 7 days for up to 3 weeks. Tumor volume was calculated by the modified ellipsoidal formula (length × width × width)/2. The experiment procedure and protocol was approved by Institutional Animal Care and Use Committee in National Cancer Center of Korea (NCC-16-231).

### Immunohistochemical staining

Formaldehyde-fixed paraffin-embedded tissue section was used for immunohistochemical staining performed with Discovery XT (Ventana Medical Systems, Tucson, AZ, USA) using anti-FAM188B antibody (Atlas Antibodies AB, Stockholm, Sweden). Parallel sections incubated with normal IgG antibody instead of primary antibodies were used as NCs.

### Statistical analysis

The statistical significance of differences between groups was determined using *χ*^2^-test and Student’s *t*-test. *p*-values <0.05 were considered statistically significant.

## Electronic supplementary material

Supplementary Figures

Supplementary Tables
