# Supplementary material for: FAM188B enhances cell survival via interaction with USP7
Source: Cell Death Dis. 2018 May 24;9(6):633. doi: 10.1038/s41419-018-0650-6 (PMC5967306; doi:10.1038/s41419-018-0650-6)
Supplement: Supplementary file 1 — Supplementary Figures [file 41419_2018_650_MOESM1_ESM.docx]

**Supplementary Information**

**
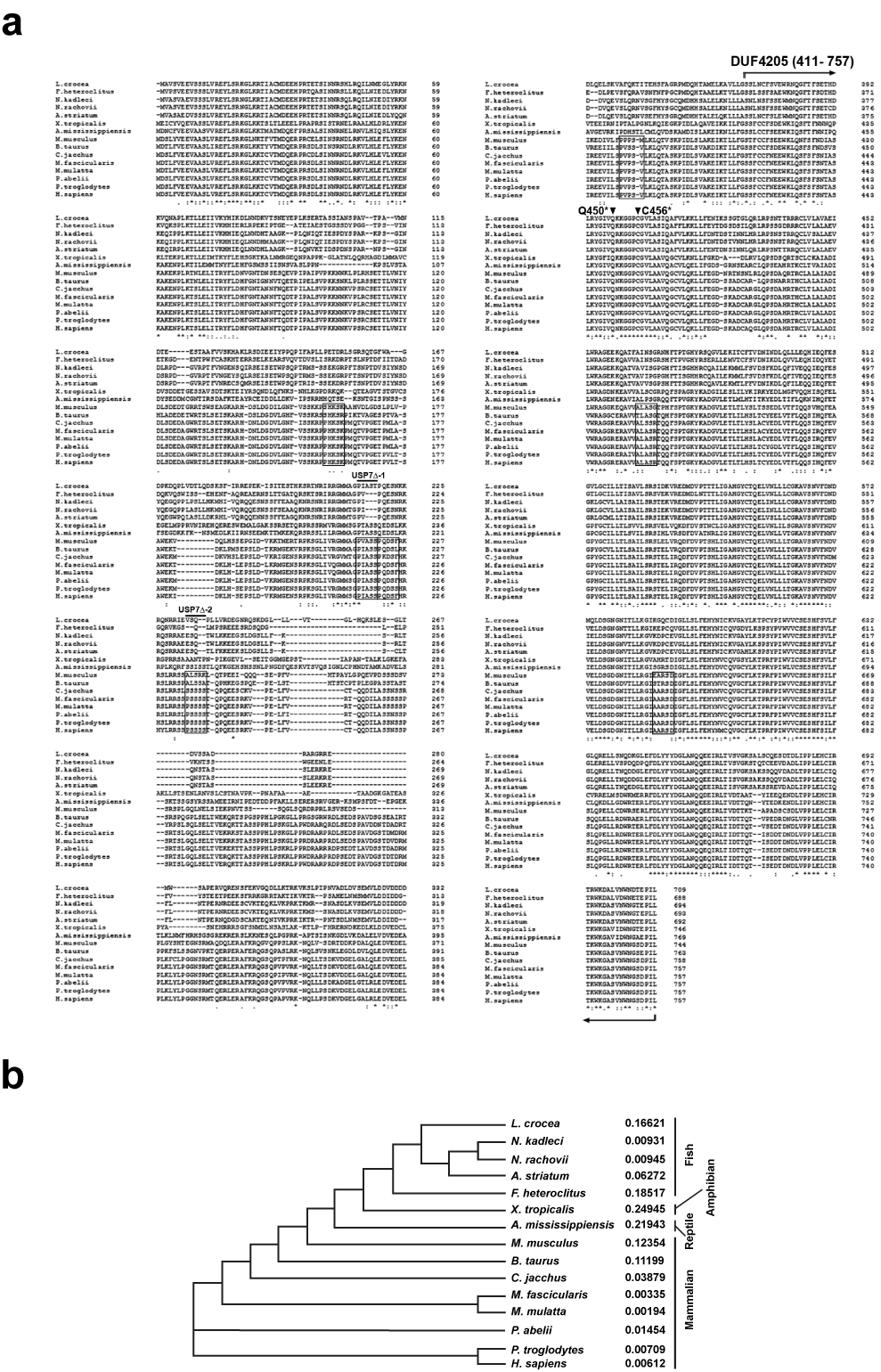
**

**Supplementary Figure 1.** Conservation of FAM188B proteins in different organisms and evolutionary distance. (**a**) Sequence alignment of FAM188B from different organisms is shown. Boxes indicate the USP7 binding motifs. Arrowheads indicate catalytic domain of MINDY DUB family. Amino acid sequence of *Larimichthys crocea*, *Nothobranchius kadleci*, *Nothobranchius rachovii*, *Aphyosemion striatum*, *Fundulus heteroclitus*, *Xenopus tropicalis*, *Alligator mississippiensis*, *Mus musculus*, *Bos taurus*, *Callithrix jacchus*, *Macaca fascicularis*, *Macaca mulatta*, *Pongo abelii*, *Pan troglodytes*, and *Homo sapiens* were obtained from GenBank^1^. Sequences were aligned software (Supplementary Figure 1a) and the genetic distances among them were calculated and cladogram was generated using Clustal Omega software^2^ (**b**) Cladogram of FAM188B based on sequence alignment.

**References**

1. Benson DA, *et al*. GenBank. Nucleic Acids Res 2014, 42(Database issue): D32-37.

2. Li W, *et al*. The EMBL-EBI bioinformatics web and programmatic tools framework. Nucleic Acids Res 2015, 43(W1): W580-584.

**
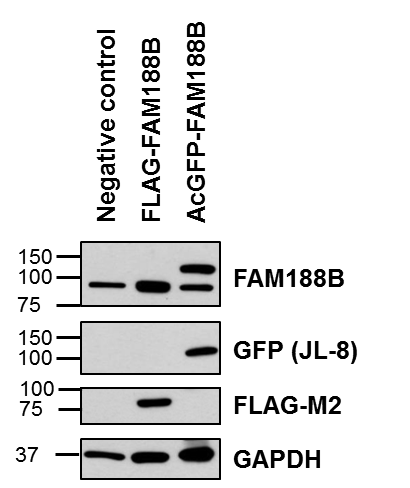
**

**Supplementary Figure 2.** Specificity confirmation of FAM188B poly-clonal antibody. The FAM188B antibody detects bands at original molecular weight (84 kDa) and AcGFP-tagged FAM188B at 113 kDa from HEK-293 cells transfected with expression plasmids. Each FLAG- and AcGFP-tagged FAM188B proteins are detected only by their specific antibody (anti-FLAG-M2 antibody for FLAG-tagged protein and JL-8 for AcGFP-tagged protein).


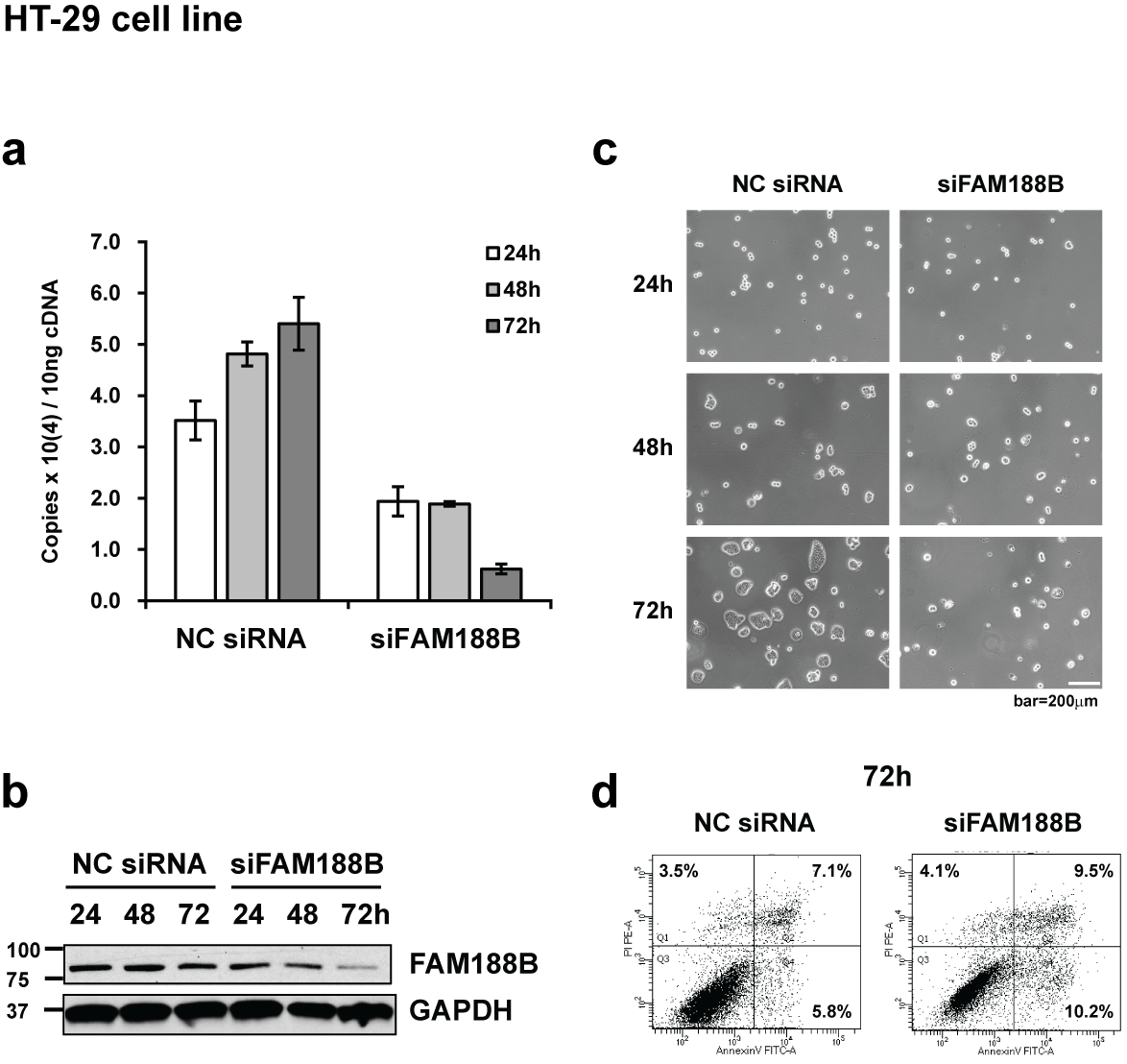


**Supplementary Figure 3.** Effect of the FAM188B expression silencing in HT-29 colon cancer cells. (**a-b**) Down-regulated FAM188B mRNA expression (**a**) and protein expression (**b**) were measured by quantitative real-time PCR and Western blot, respectively. (**c**) Cells transfected with NC siRNA or siFAM188B were observed for 72 h. Scale bar = 200μm (**d**) Annexin V and PI were measured by Flow cytometry for apoptosis percentage after NC siRNA or siFAM188B treatment (PI stained cells apoptosis population: UR and UL).


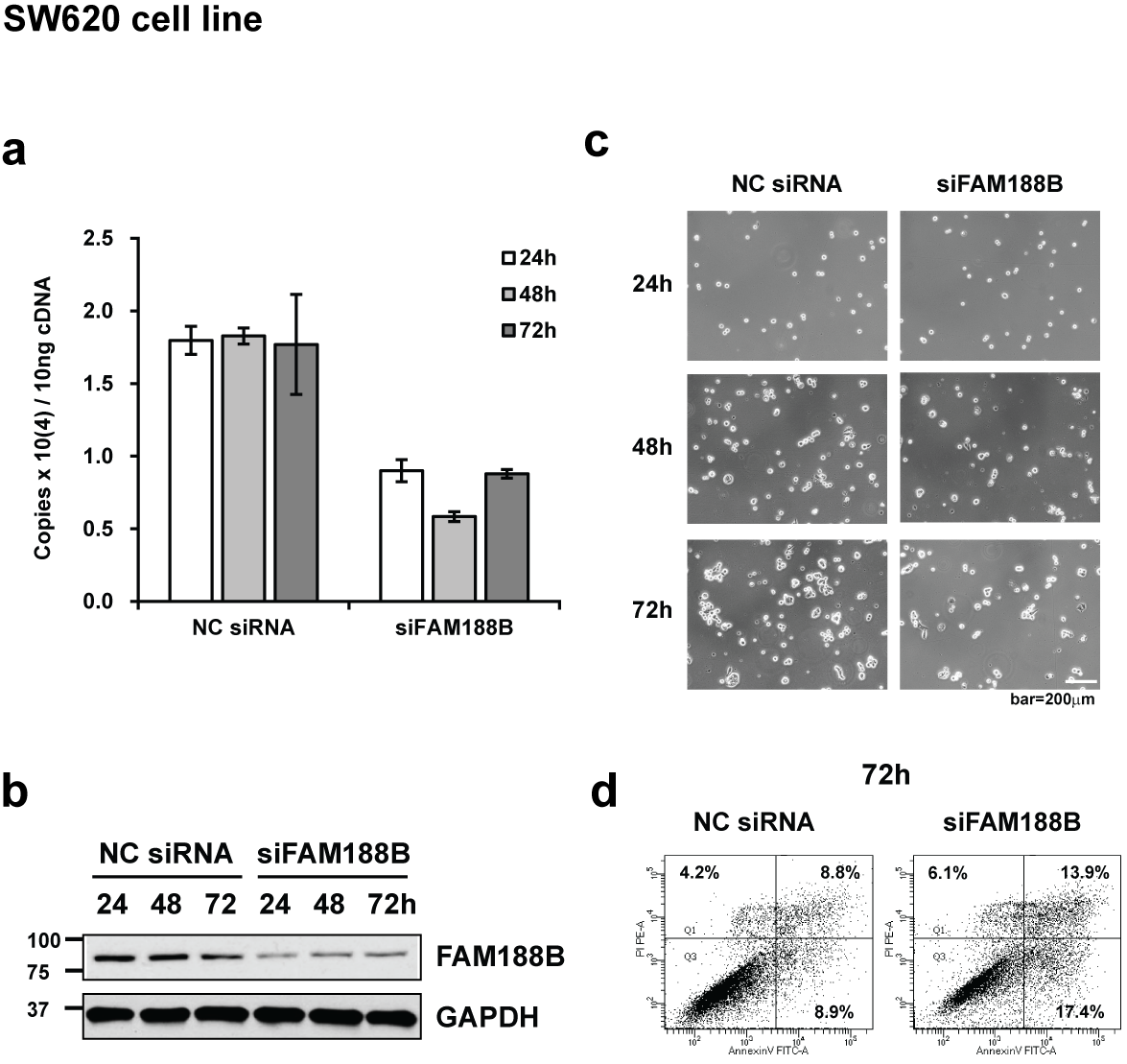


**Supplementary Figure 4.** Effect of the FAM188B expression silencing in SW620 colon cancer cells. (**a-b**) Down-regulated FAM188B mRNA expression (**a**) and protein expression (**b**) were measured by quantitative real-time PCR and Western blot, respectively. (**c**) Cells transfected with NC siRNA or siFAM188B were observed for 72 h. Scale bar = 200μm (**d**) Annexin V and PI were measured by Flow cytometry for apoptosis percentage after NC siRNA or siFAM188B treatment (PI stained cells apoptosis population: UR and UL).


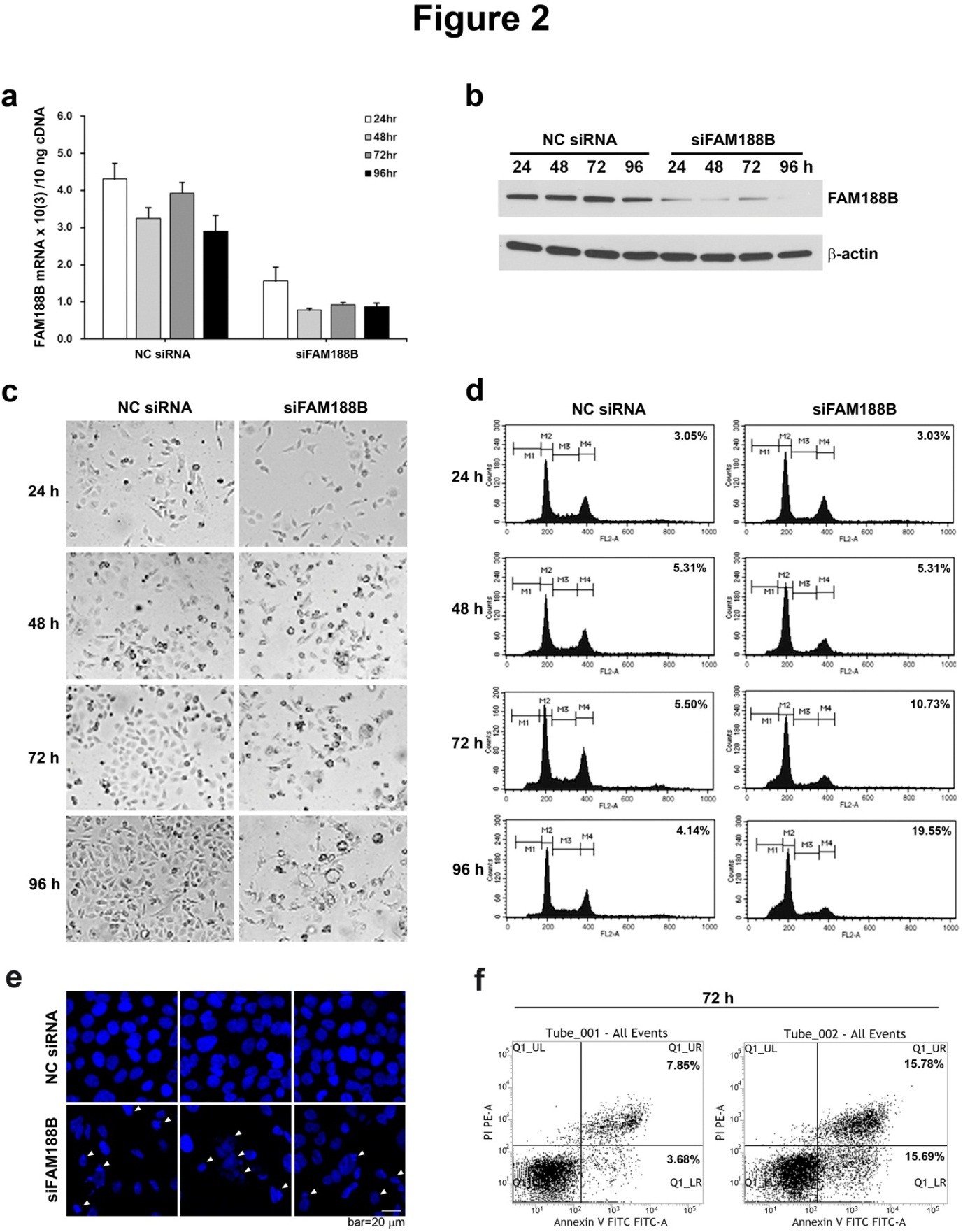


**Supplementary Figure 5.** Effect of the FAM188B expression silencing in AGS gastric cancer cells. (**a-b**) Down-regulated FAM188B mRNA expression (**a**) and protein expression (**b**) were measured by quantitative real-time PCR and Western blot, respectively. (**c**) Cells transfected with NC siRNA or siFAM188B were observed for 96 h. (**d**) PI were measured by Flow cytometry for apoptosis percentage after siRNA treatment. (**e**) Nuclear fragmentation was observed in siFAM188B treated AGS cells. (**f**) Annexin V assay showed increased dead population of AGS cell with siFAM188B treatment (PI stained cells apoptosis population: UR and UL).

**a**


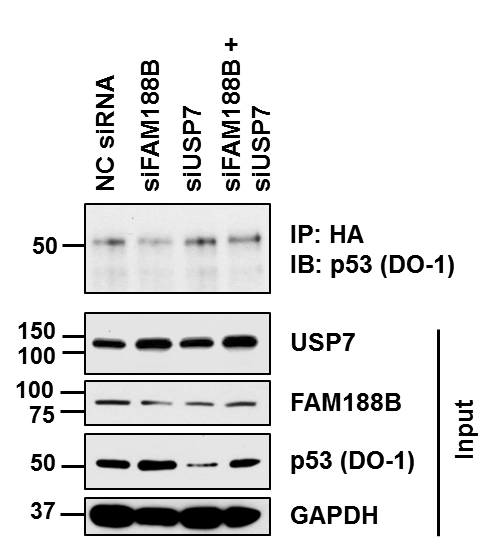


**b**


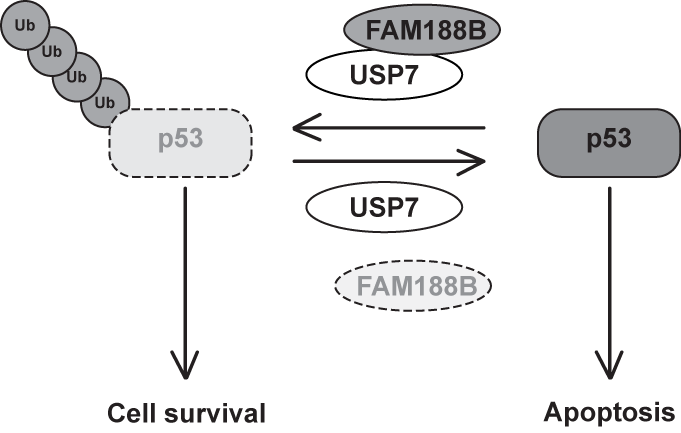


**Supplementary Figure 6.** A proposed model for regulation of p53 stability by FAM188B and USP7. **(a)** Ubiquitinated p53 is decreased by siFAM188B treatment, but increased when siUSP7 is treated. The additional treatment of siFAM188B restored the p53 level. **(b)** FAM188B interact with USP7 and make p53 unstable in cancer cells, but when FAM188B is knocked-down, p53 becomes stable by deubiquitination by USP7 and activated.
