# Supplementary material for: FAM188B enhances cell survival via interaction with USP7
Source: Cell Death Dis. 2018 May 24;9(6):633. doi: 10.1038/s41419-018-0650-6 (PMC5967306; doi:10.1038/s41419-018-0650-6)
Supplement: Supplementary file 2 — Supplementary Tables [file 41419_2018_650_MOESM2_ESM.docx]

**Supplementary Table 1.** Primer sequences for PCR reactions

| **Target name** | **Forward primer (5’ – 3’)**  **Reverse primer (5’ – 3’)** | **Amplicon size (bp)** | **Annealing temp. (°C)** | **Reference** |
| --- | --- | --- | --- | --- |
| FAM188B | GAGTCCTGGCAGCTGTCCAA | 97 | 54 | In house design |
|  | GCATCTGAAGGCTGCAGTCC |  |  |  |
|  |  |  |  |  |
| ACTB | CATCGAGCACGGCATCGTCA | 211 | 58 |  |
|  | TAGCACAGCCTGGATAGCAAC |  |  |  |
|  |  |  |  |  |
| BAX promoter_p53RE | TTGGGCTCACAAGTTAGAGACAAG | 154 | 60 | [37] |
|  | CCTGGATCTAGCAATATAGCCCAC |  |  |  |
|  |  |  |  |  |
| PUMA promoter_p53RE | TCAGTGTGTGTGTCCGACTGTC | 210 | 60 | [37] |
|  | GGCAGGGCCTAGCCCA |  |  |  |

**Supplementary Table 2.** Functional classification of FAM188B interacting proteins (97/104) by Biological process of Gene Ontology.

| **Term** | **Count** | **%** | **P-Value** | **Genes** |
| --- | --- | --- | --- | --- |
| GO:0006414~translational elongation | 9 | 9.3 | 2.39E-07 | EF1G_HUMAN, RLA2_HUMAN, RL10_HUMAN, RL13A_HUMAN, RL22_HUMAN, RL4_HUMAN, RS6_HUMAN, RL21_HUMAN, RL14_HUMAN |
| GO:0006412~translation | 13 | 13.4 | 1.14E-06 | EF1G_HUMAN, EIF3H_HUMAN, RL10_HUMAN, RL13A_HUMAN, EIF3A_HUMAN, RL22_HUMAN, RL4_HUMAN, RS6_HUMAN, RL14_HUMAN, EIF3E_HUMAN, RLA2_HUMAN, EIF3B_HUMAN, RL21_HUMAN |
| GO:0016071~mRNA metabolic process | 12 | 12.4 | 2.15E-05 | RU2A_HUMAN, PRP8_HUMAN, RPB2_HUMAN, EIF3E_HUMAN, SF3B1_HUMAN, SF3B4_HUMAN, HSP71_HUMAN, SRRM2_HUMAN, PRP19_HUMAN, PAIRB_HUMAN, PM14_HUMAN, HNRPF_HUMAN |
| GO:0008380~RNA splicing | 10 | 10.3 | 7.80E-05 | RU2A_HUMAN, 2AAA_HUMAN, PRP8_HUMAN, RPB2_HUMAN, SF3B1_HUMAN, SF3B4_HUMAN, SRRM2_HUMAN, PRP19_HUMAN, PM14_HUMAN, HNRPF_HUMAN |
| GO:0007398~ectoderm development | 8 | 8.2 | 2.70E-04 | K1C15_HUMAN, K1C10_HUMAN, K22E_HUMAN, K2C6A_HUMAN, K1C13_HUMAN, K1C9_HUMAN, K2C1_HUMAN, DESP_HUMAN |
| GO:0000398~nuclear mRNA splicing, via spliceosome | 7 | 7.2 | 4.24E-04 | RU2A_HUMAN, PRP8_HUMAN, RPB2_HUMAN, SF3B1_HUMAN, SF3B4_HUMAN, PM14_HUMAN, HNRPF_HUMAN |
| GO:0000377~RNA splicing, via transesterification reactions with bulged adenosine as nucleophile | 7 | 7.2 | 4.24E-04 | RU2A_HUMAN, PRP8_HUMAN, RPB2_HUMAN, SF3B1_HUMAN, SF3B4_HUMAN, PM14_HUMAN, HNRPF_HUMAN |
| GO:0000375~RNA splicing, via transesterification reactions | 7 | 7.2 | 4.24E-04 | RU2A_HUMAN, PRP8_HUMAN, RPB2_HUMAN, SF3B1_HUMAN, SF3B4_HUMAN, PM14_HUMAN, HNRPF_HUMAN |
| GO:0006396~RNA processing | 12 | 12.4 | 6.69E-04 | RU2A_HUMAN, 2AAA_HUMAN, PRP8_HUMAN, RPB2_HUMAN, SF3B1_HUMAN, SF3B4_HUMAN, SRRM2_HUMAN, PRP19_HUMAN, PM14_HUMAN, RS6_HUMAN, RL14_HUMAN, HNRPF_HUMAN |
| GO:0006457~protein folding | 7 | 7.2 | 9.12E-04 | HS74L_HUMAN, TCPB_HUMAN, BAG2_HUMAN, RUVB2_HUMAN, TCPQ_HUMAN, GRP75_HUMAN, PPIB_HUMAN |
| GO:0006397~mRNA processing | 9 | 9.3 | 9.80E-04 | RU2A_HUMAN, PRP8_HUMAN, RPB2_HUMAN, SF3B1_HUMAN, SF3B4_HUMAN, SRRM2_HUMAN, PRP19_HUMAN, PM14_HUMAN, HNRPF_HUMAN |
| GO:0008544~epidermis development | 7 | 7.2 | 1.11E-03 | K1C15_HUMAN, K1C10_HUMAN, K22E_HUMAN, K1C13_HUMAN, K1C9_HUMAN, K2C1_HUMAN, DESP_HUMAN |
| GO:0006413~translational initiation | 4 | 4.1 | 2.90E-03 | EIF3E_HUMAN, EIF3H_HUMAN, EIF3B_HUMAN, EIF3A_HUMAN |
| GO:0007017~microtubule-based process | 7 | 7.2 | 5.45E-03 | KIF11_HUMAN, NPM_HUMAN, PCM1_HUMAN, IMA2_HUMAN, KIF7_HUMAN, LPPRC_HUMAN, TBB6_HUMAN |
| GO:0009303~rRNA transcription | 3 | 3.1 | 5.72E-03 | P53_HUMAN, TCOF_HUMAN, TF3C1_HUMAN |
| GO:0051297~centrosome organization | 3 | 3.1 | 1.75E-02 | KIF11_HUMAN, NPM_HUMAN, PCM1_HUMAN |
| GO:0070647~protein modification by small protein conjugation or removal | 5 | 5.2 | 1.89E-02 | CDC73_HUMAN, USP9X_HUMAN, UBP7_HUMAN, CTR9_HUMAN, PRP19_HUMAN |
| GO:0031023~microtubule organizing center organization | 3 | 3.1 | 2.07E-02 | KIF11_HUMAN, NPM_HUMAN, PCM1_HUMAN |
| GO:0042149~cellular response to glucose starvation | 2 | 2.1 | 2.52E-02 | P53_HUMAN, GRP78_HUMAN |
| GO:0022402~cell cycle process | 9 | 9.3 | 2.70E-02 | KIF11_HUMAN, RAD50_HUMAN, NPM_HUMAN, IMA2_HUMAN, USP9X_HUMAN, APC7_HUMAN, P53_HUMAN, PPP6_HUMAN, APC1_HUMAN |
| GO:0006446~regulation of translational initiation | 3 | 3.1 | 2.79E-02 | EIF3E_HUMAN, EIF3H_HUMAN, EIF3B_HUMAN |
| GO:0007018~microtubule-based movement | 4 | 4.1 | 3.52E-02 | KIF11_HUMAN, KIF7_HUMAN, LPPRC_HUMAN, TBB6_HUMAN |
| GO:0051052~regulation of DNA metabolic process | 4 | 4.1 | 3.60E-02 | RAD50_HUMAN, 2AAA_HUMAN, IMA2_HUMAN, P53_HUMAN |
| GO:0007252~I-kappaB phosphorylation | 2 | 2.1 | 3.75E-02 | PRDX4_HUMAN, ELP1_HUMAN |
| GO:0070271~protein complex biogenesis | 8 | 8.2 | 4.15E-02 | NDUS7_HUMAN, NPM_HUMAN, 2AAA_HUMAN, RPB2_HUMAN, P53_HUMAN, RO52_HUMAN, TBB6_HUMAN, ELP1_HUMAN |
| GO:0006461~protein complex assembly | 8 | 8.2 | 4.15E-02 | NDUS7_HUMAN, NPM_HUMAN, 2AAA_HUMAN, RPB2_HUMAN, P53_HUMAN, RO52_HUMAN, TBB6_HUMAN, ELP1_HUMAN |
| GO:0010390~histone monoubiquitination | 2 | 2.1 | 4.37E-02 | CDC73_HUMAN, CTR9_HUMAN |
| GO:0051095~regulation of helicase activity | 2 | 2.1 | 4.37E-02 | P53_HUMAN, SSBP_HUMAN |
| GO:0022403~cell cycle phase | 7 | 7.2 | 4.81E-02 | KIF11_HUMAN, RAD50_HUMAN, IMA2_HUMAN, USP9X_HUMAN, APC7_HUMAN, PPP6_HUMAN, APC1_HUMAN |
| GO:0033523~histone H2B ubiquitination | 2 | 2.1 | 4.98E-02 | CDC73_HUMAN, CTR9_HUMAN |
| GO:0006513~protein monoubiquitination | 2 | 2.1 | 5.58E-02 | CDC73_HUMAN, CTR9_HUMAN |
| GO:0060429~epithelium development | 5 | 5.2 | 5.66E-02 | K22E_HUMAN, IF172_HUMAN, K2C4_HUMAN, K2C3_HUMAN, DESP_HUMAN |
| GO:0030855~epithelial cell differentiation | 4 | 4.1 | 5.67E-02 | K22E_HUMAN, K2C4_HUMAN, K2C3_HUMAN, DESP_HUMAN |
| GO:0007049~cell cycle | 10 | 10.3 | 5.76E-02 | CDC73_HUMAN, KIF11_HUMAN, RAD50_HUMAN, NPM_HUMAN, IMA2_HUMAN, USP9X_HUMAN, APC7_HUMAN, P53_HUMAN, PPP6_HUMAN, APC1_HUMAN |
| GO:0007005~mitochondrion organization | 4 | 4.1 | 5.77E-02 | NDUS7_HUMAN, TIM50_HUMAN, P53_HUMAN, SSBP_HUMAN |
| GO:0000279~M phase | 6 | 6.2 | 5.84E-02 | KIF11_HUMAN, RAD50_HUMAN, IMA2_HUMAN, USP9X_HUMAN, APC7_HUMAN, APC1_HUMAN |
| GO:0007010~cytoskeleton organization | 7 | 7.2 | 5.90E-02 | FHL3_HUMAN, KIF11_HUMAN, NPM_HUMAN, PCM1_HUMAN, K2C4_HUMAN, K2C3_HUMAN, K1C9_HUMAN |
| GO:0065003~macromolecular complex assembly | 9 | 9.3 | 6.04E-02 | NDUS7_HUMAN, NPM_HUMAN, 2AAA_HUMAN, RPB2_HUMAN, P53_HUMAN, RO52_HUMAN, EIF3A_HUMAN, TBB6_HUMAN, ELP1_HUMAN |
| GO:0006983~ER overload response | 2 | 2.1 | 6.18E-02 | P53_HUMAN, GRP78_HUMAN |
| GO:0042273~ribosomal large subunit biogenesis | 2 | 2.1 | 6.18E-02 | NPM_HUMAN, RL14_HUMAN |
| GO:0042274~ribosomal small subunit biogenesis | 2 | 2.1 | 6.78E-02 | NPM_HUMAN, RS6_HUMAN |
| GO:0006511~ubiquitin-dependent protein catabolic process | 5 | 5.2 | 6.82E-02 | USP9X_HUMAN, UBP7_HUMAN, APC7_HUMAN, UBR5_HUMAN, APC1_HUMAN |
| GO:0033554~cellular response to stress | 8 | 8.2 | 6.83E-02 | RAD50_HUMAN, DHRS2_HUMAN, P53_HUMAN, TOP2A_HUMAN, UBR5_HUMAN, RUVB2_HUMAN, GRP78_HUMAN, PRP19_HUMAN |
| GO:0042981~regulation of apoptosis | 10 | 10.3 | 6.88E-02 | NPM_HUMAN, 2AAA_HUMAN, DHRS2_HUMAN, P53_HUMAN, TOP2A_HUMAN, HSP71_HUMAN, GRP78_HUMAN, IFT57_HUMAN, GRP75_HUMAN, RS6_HUMAN |
| GO:0043067~regulation of programmed cell death | 10 | 10.3 | 7.23E-02 | NPM_HUMAN, 2AAA_HUMAN, DHRS2_HUMAN, P53_HUMAN, TOP2A_HUMAN, HSP71_HUMAN, GRP78_HUMAN, IFT57_HUMAN, GRP75_HUMAN, RS6_HUMAN |
| GO:0010941~regulation of cell death | 10 | 10.3 | 7.36E-02 | NPM_HUMAN, 2AAA_HUMAN, DHRS2_HUMAN, P53_HUMAN, TOP2A_HUMAN, HSP71_HUMAN, GRP78_HUMAN, IFT57_HUMAN, GRP75_HUMAN, RS6_HUMAN |
| GO:0043066~negative regulation of apoptosis | 6 | 6.2 | 7.48E-02 | NPM_HUMAN, DHRS2_HUMAN, P53_HUMAN, HSP71_HUMAN, GRP78_HUMAN, GRP75_HUMAN |
| GO:0006913~nucleocytoplasmic transport | 4 | 4.1 | 7.71E-02 | NPM_HUMAN, IMA2_HUMAN, P53_HUMAN, GRP75_HUMAN |
| GO:0043069~negative regulation of programmed cell death | 6 | 6.2 | 7.84E-02 | NPM_HUMAN, DHRS2_HUMAN, P53_HUMAN, HSP71_HUMAN, GRP78_HUMAN, GRP75_HUMAN |
| GO:0060548~negative regulation of cell death | 6 | 6.2 | 7.91E-02 | NPM_HUMAN, DHRS2_HUMAN, P53_HUMAN, HSP71_HUMAN, GRP78_HUMAN, GRP75_HUMAN |
| GO:0051169~nuclear transport | 4 | 4.1 | 7.94E-02 | NPM_HUMAN, IMA2_HUMAN, P53_HUMAN, GRP75_HUMAN |
| GO:0043933~macromolecular complex subunit organization | 9 | 9.3 | 8.15E-02 | NDUS7_HUMAN, NPM_HUMAN, 2AAA_HUMAN, RPB2_HUMAN, P53_HUMAN, RO52_HUMAN, EIF3A_HUMAN, TBB6_HUMAN, ELP1_HUMAN |
| GO:0000278~mitotic cell cycle | 6 | 6.2 | 8.65E-02 | KIF11_HUMAN, IMA2_HUMAN, USP9X_HUMAN, APC7_HUMAN, PPP6_HUMAN, APC1_HUMAN |
| GO:0051276~chromosome organization | 7 | 7.2 | 8.81E-02 | CDC73_HUMAN, RAD50_HUMAN, NPM_HUMAN, P53_HUMAN, TOP2A_HUMAN, CTR9_HUMAN, RUVB2_HUMAN |
| GO:0006974~response to DNA damage stimulus | 6 | 6.2 | 8.88E-02 | RAD50_HUMAN, P53_HUMAN, TOP2A_HUMAN, UBR5_HUMAN, RUVB2_HUMAN, PRP19_HUMAN |
| GO:0044265~cellular macromolecule catabolic process | 9 | 9.3 | 8.94E-02 | EIF3E_HUMAN, USP9X_HUMAN, UBP7_HUMAN, APC7_HUMAN, VPRBP_HUMAN, UBR5_HUMAN, HSP71_HUMAN, PJA2_HUMAN, APC1_HUMAN |
| GO:0043281~regulation of caspase activity | 3 | 3.1 | 8.99E-02 | P53_HUMAN, GRP78_HUMAN, IFT57_HUMAN |
| GO:0052548~regulation of endopeptidase activity | 3 | 3.1 | 9.58E-02 | P53_HUMAN, GRP78_HUMAN, IFT57_HUMAN |
| GO:0043086~negative regulation of catalytic activity | 5 | 5.2 | 9.99E-02 | 2AAA_HUMAN, APC7_HUMAN, P53_HUMAN, GRP78_HUMAN, APC1_HUMAN |
